# Strengthening Kenya's public health response to reproductive coercion and intimate partner violence in family planning clinics: applying the FRAME + IS approach

**DOI:** 10.3389/frph.2025.1630877

**Published:** 2026-01-05

**Authors:** Jamie Menzel, Jasmine Uysal, Erin Pearson, Jane Namwebya, Mary Gathitu, Alice Mwangangi, Clarice Okumu, Betty Chirchir, Wilson Liambila, George Odwe, Edward Serem, Chi-Chi Undie, Jay Silverman

**Affiliations:** 1 Center on Gender Equity and Health, Division of Infectious Diseases and Global Public Health, School of Medicine, University of California, San Diego, CA, United States; 2 Population Council Kenya, Nairobi, Kenya; 3 Division of Reproductive, Maternal, Newborn, Child and Adolescent Health, Ministry of Health, Nairobi, Kenya; 4 Department of Health, Eldoret, Kenya; 5 Population Council, Inc., Nairobi, Kenya; 6 Celia Scott Weatherhead School of Public Health and Tropical Medicine, Tulane University, New Orleans, LA, United States

**Keywords:** family planning, gender-based violence, reproductive coercion, intimate partner violence, evidence-based intervention, adaptation, Kenya, institutionalization

## Abstract

**Background:**

Reproductive coercion (RC) and intimate partner violence (IPV) undermine reproductive autonomy and are prevalent among women seeking family planning (FP) services. In response, Kenya's Ministry of Health (MOH) selected ARCHES (Addressing Reproductive Coercion in Health Settings), an evidence-based intervention (EBI) integrating universal education, screening, and support on RC and IPV during routine FP counseling, for national adaptation and scale-up within a hybrid implementation-effectiveness trial. Institutionalizing such interventions within public health systems requires careful adaptation to ensure contextual fit while preserving core functions.

**Methods:**

We developed and applied FRAME + IS, a unified adaptation-tracking framework that integrates the FRAME and FRAME-IS tools, to systematically document modifications made to the ARCHES intervention and its implementation strategies. The adaptation process was guided by adaptive management and the ADAPT-ITT framework and included formative research, national and county-level workshops, iterative piloting, and implementation planning, led by the Kenya MOH.

**Results:**

We identified 12 key adaptations: six related to intervention content and six related to implementation strategies. Most were planned (75%) and occurred prior to implementation (83%). Adaptations addressed feasibility, sustainability, and alignment with government systems. Examples include integration into national FP counseling protocols, namely the Balanced Counseling Strategy Plus, a shift from paper-based tools to a mobile app, and a formalized provider mentorship schedule. While the majority of adaptations were consistent with the original ARCHES intervention core strategies (58%), several, including removal of discreet contraceptive use counseling from official provider training materials and job-aids, were not consistent with the original model and reflected necessary trade-offs due to political sensitivities and implementation realities. The Kenya MOH was the final decision-maker on all adaptations, incorporating input from national and county-level staff, providers, and intervention experts.

**Conclusion:**

This is the first published example of a government adopting provider training and guidelines to integrate RC and IPV response within FP services while systematically tracking these adaptations within a public health system. By applying FRAME + IS, this study offers both a practical roadmap for governments seeking to institutionalize IPV and RC interventions at scale and a streamlined framework to document changes to EBIs and implementation strategies during complex integration processes.

## 1 Introduction

Reproductive coercion (RC) ‒ behaviors that seek to control women's contraceptive and pregnancy decisions (1) ‒ and intimate partner violence (IPV) ‒ abuse by a former or current intimate partner (2) ‒ are both forms of gender-based violence (GBV) consistently linked with adverse reproductive outcomes among women, including unintended pregnancy and HIV (3–5). Globally, over one in four ever-partnered women report experiencing IPV in their lifetime and more than one in ten in the past year (6), with rates rising closer to one in three women for Sub-Saharan Africa. While global RC estimates are lacking, a multi-country study in 10 low- and middle-income countries (LMICs), primarily in Sub-Saharan Africa, found prevalence ranging from 3.1% in Niger to 20.3% in the Democratic Republic of the Congo (7). In Kenya, the 2022 Demographic and Health Survey found 41% of women report IPV (8) and 11% report pressure to become pregnant by partners or family members (9), compared to 7% by partners only among married women from Kenya Performance Monitoring for Action (PMA2020) data (7). RC and IPV have been found to be particularly common among women seeking family planning (FP) services (10, 11). Among our sample of FP clients in Uasin Gishu County, Kenya, we found an overall RC prevalence of 42% by partners or family members (12), underscoring the urgent need for integration of RC and IPV screening and support into Kenya's FP system.

In 2019 the World Health Organization (WHO) released a training curriculum to build providers capacity to screen and address women's common experiences of RC and IPV within routine FP services (13). Yet, to date, attempts to systematically integrate RC and IPV screening into government FP systems have been both sparse and suboptimal. However, institutionalizing screening and response to RC and IPV is critical to advancing women's health and rights. One pathway for government health systems to successfully institutionalize RC and IPV screening and response is to adapt and scale existing evidence-based interventions (EBIs) within the public FP system (14–17).

One such EBI is Addressing Reproductive Coercion in Health Care Settings (ARCHES), which was developed to address and respond to women's experience of RC and IPV within FP services (18, 19). ARCHES trains existing service providers to integrate strategies to address and respond to RC and IPV into standard FP counseling protocols for all female clients whom they are able to counsel privately (20, 21). ARCHES was originally developed and tested in the United States (18, 19, 21) by a team of researchers in partnership with the non-profit Futures Without Violence. Since then, the model has been successfully adapted to and implemented in private FP clinics in Nairobi and abortion care facilities in Bangladesh (22, 23), where it improved contraceptive uptake and continued use, increased women's confidence in controlling contraceptive decisions in the face of opposition, reduced incident pregnancy, enhanced IPV support service awareness, and reduced IPV (24–27). In line with Kenya's commitment to address unintended pregnancy and GBV (28), the Kenya Ministry of Health (MOH) selected ARCHES for scale-up within public sector FP services.

Large public health systems, particularly in LMICs, are almost always more complex than the controlled settings in which interventions were originally tested, which can pose challenges for institutionalization and scale-up (29, 30). For family planning, some of these challenges can include contraceptive stockouts, privacy concerns, restrictive social norms, overburdened providers, and political and financial constraints (31–35). In Kenya, just over 60% of women receive their contraceptive methods from public service delivery points (9), which frequently struggle with challenges in contraceptive stockouts (36, 37), provider-imposed barriers to contraceptive methods (38), and quality of care and privacy issues, particularly for younger women (39, 40). Private pharmacies and clinics play a complementary role, especially for younger women and those seeking short-term methods, as they are often perceived as more discreet (i.e., able to be used without a husband or family member knowing) or convenient (38, 41). Despite increased convenience of private facilities for some women, the public health system continues to bear the greatest burden for method provision which has expanded access and use to reduce unmet need across Kenya (41).

Given the challenges that exist within the public health system in Kenya and elsewhere, institutionalization within these settings nearly always requires some degree of adaptation (42) – modifying interventions to fit new contexts while maintaining effectiveness (43). Adaptation can involve modifications to the content or delivery of an intervention (e.g., language, format, or length) (44) and/or the implementation strategies used to support adoption, implementation, and sustainability of an intervention in the real-world (e.g., training, monitoring, policy integration, and stakeholder engagement) (45). Excessive modifications, however, risk unintended “drift” from the core program components, potentially undermining effectiveness or sustainability (46, 47) and undermines ability to compare effectiveness and implementation across adaptations and contexts. Systematic tracking is essential to document adaptations, assess their impact, and promote fidelity.

This paper applies a new integrated adaptation-tracking approach – the Framework for Reporting Adaptations and Modifications-Enhanced plus Implementation Strategies (FRAME + IS) – which combines the original FRAME (44) and FRAME-IS (45) frameworks. We use FRAME + IS to systematically and rigorously document key modifications made to the ARCHES intervention and its implementation strategies in collaboration with the Kenya MOH through a mixed methods approach, as part of a hybrid implementation-effectiveness trial. Our analysis captures both planned and spontaneous adaptations across system, organizational, provider, and individual levels as part of a broader effort to support national scale-up. To our knowledge, this is the first published example of a government adopting provider training and guidelines to integrate RC and IPV response within FP services while systematically tracking these adaptations within a public health system. By applying FRAME + IS, this study offers both a practical roadmap for governments seeking to institutionalize IPV and RC interventions at scale and a streamlined framework to document changes to EBIs and implementation strategies during complex integration processes.

## 2 Methods

### 2.1 The ARCHES approach and adaptation in Kenya

ARCHES is an integrated intervention designed to be embedded within routine FP counseling that trains providers to recognize and respond to RC and IPV. The approach is anchored in three core strategies:

A. provision of information and screening on RC and strategies for discreet contraceptive method use;
B. information and screening on IPV with supported referrals for those who disclose; and
C. distribution of an easily concealable, rights-based educational mini-booklet that includes information on RC, IPV, contraceptive options, and locally available IPV support services (20, 21).

Unlike traditional GBV screening models that rely on client disclosure, ARCHES employs a universal education and support approach. All female FP clients whom providers can counsel privately (without anyone else present) are offered ARCHES content. Discreet contraceptive method use strategies are integrated into standard method counseling, presented alongside side effects and contraindications. IPV screening typically follows RC screening once rapport is established. All clients are offered the mini-booklet, and clients who disclose IPV are offered an optional immediate phone-based connection to local services but are never pressured to accept support or materials. Providers are also trained on the values of women-centered care where clients' voices and desires are prioritized above all others.

ARCHES was initially adapted to a LMIC setting in Nairobi, Kenya through collaboration with a private FP non-profit Family Health Options of Kenya (FHOK), the International Planned Parenthood Federation (IPPF), and Population Council Kenya from 2016 to 2019. Formative research among clients, providers, and clinic administrators informed the initial adaptation, followed by pretesting and a three-month pilot to refine feasibility and acceptability. For this Nairobi adaptation of ARCHES, the model was integrated into the GATHER (Greet, Ask, Tell, Help, Explain, Return) approach to FP counseling (48), which was the standard of practice at FHOK clinics. Overall, core ARCHES components were retained from the original U.S.-based model, but several adaptations addressed contextual challenges. These included developing new protocols to ensure privacy with clients away from accompanying male partners or family members to deliver RC/IPV content; offering women the option to bring male partners or family members back to the clinic for more information on FP (without pressure to do so); enhanced provider training on RC, IPV, and women-centered care to improve provider attitudes; enhanced post-training provider mentorship; and tools and materials tailored to the FHOK health system.

Provider training in the initial Nairobi adaptation of ARCHES was expanded from a half day to a three-day format grounded in Social Learning Theory (49), led by master trainers from clinics, researchers, and FHOK leadership. The curriculum includes structured content on RC and IPV, values clarification activities, engagement with the ARCHES counseling strategies, and practice with peers and supervisory feedback. Providers were supported post-training through mentorship, supervision, and monthly peer learning meetings. Mentorship sessions were provided from one to three times per week in-clinic depending on facility performance, assessed via client exit interviews, for six months post-training. Counseling rooms were equipped with visual job aids and rights-based posters, and providers used a short one-page job aid (see Supplementary Data Sheet 1) and a provider-client flipbook that integrates ARCHES content with standard FP method counseling.

Once the Nairobi model was refined, a six-month non-randomized controlled trial was conducted across six clinics with 649 women seeking FP care. The trial demonstrated promising outcomes: increased self-efficacy to use contraception despite RC, reduced acceptance of RC, increased awareness of IPV services, a reduction in physical IPV, and decreased incident pregnancy (26).^1^ However, the study also found significantly smaller reductions in RC and sexual IPV among ARCHES participants compared to controls. These unexpected findings may reflect increased awareness and reporting of RC and IPV among intervention participants, or possible unintended consequences of discreet contraceptive use, a harm reduction strategy supported through ARCHES, that could provoke coercion or conflict if discovered (though authors uncovered no evidence of this adverse event). Additionally, higher baseline levels of violence in intervention clinics suggest possible residual unmeasured confounding even after adjustment. Despite these complexities, the finding of a 40% relative reduction in physical IPV, increased client agency, improved pregnancy prevention, and strong collaborative engagement contributed to strong support of the model from the Kenya MOH. Recognizing the intervention's alignment with national priorities to reduce GBV and unintended pregnancy, the MOH committed to subsequently adapting and scaling the Nairobi ARCHES model for implementation within the public-sector family planning system nationally starting in Uasin Gishu county in 2021.

### 2.2 Kenya MOH adaptation process for scale-up

The subsequent Kenya MOH ARCHES adaptation process was guided and overseen by three groups: A) national-level MOH staff who made final decisions about adaptation of the model, B) an implementation team consisting of Uasin Gishu county-level MOH staff who advised the national-level MOH staff regarding implementation considerations for the adaptation, and C) a team of ARCHES intervention experts from University of California, San Diego and Tulane University including the co-designer of the model, researchers who led the adaptations and evaluations of ARCHES in Nairobi and Bangladesh, and a senior implementation science researcher with experience in adaptation for scale-up, who advised on how to maintain fidelity to the model during adaptation and promote institutionalization and sustainability of the adapted model for scale.

An adaptive management approach guided the adaptation of ARCHES, emphasizing stakeholder collaboration, continuous learning, and context-responsive decision-making. Adaptive management is a structured, evidence-based, and participatory process that engages multi-level stakeholders to iteratively refine interventions based on real-time data and evolving system needs (50). To further structure and systematize the adaptation process, we applied the ADAPT-ITT model, which provides step-by-step guidance for planning and aligning EBIs with new contexts through stakeholder engagement, iterative learning, and phased decision-making (51). The ADAPT-ITT model, originally developed for adapting HIV EBIs, consists of eight sequential phases: 1) **assessment** of the new target population, 2) **decide** on the EBI to be adapted, 3) **adaptation** using a pretesting methodology to understand what can be adopted or adapted, 4) **production** of draft 1 of the adapted EBI that balances stakeholder priorities and fidelity to core elements, 5) engage **topical experts** to provide technical assistance in substantive content areas, 6) **integration** of content from topical experts to create draft 2 of the adapted EBI and conducting readability testing to create draft 3, 7) **training** all personnel involved in implementing the adapted EBI, and 8) **testing** draft 3 of the adapted EBI (51). Although we implemented all phases of the ADAPT-ITT model, we adjusted content and order to better fit with government procedures, stakeholder preferences, and resources available for this adaptation.

**Figure 1:**
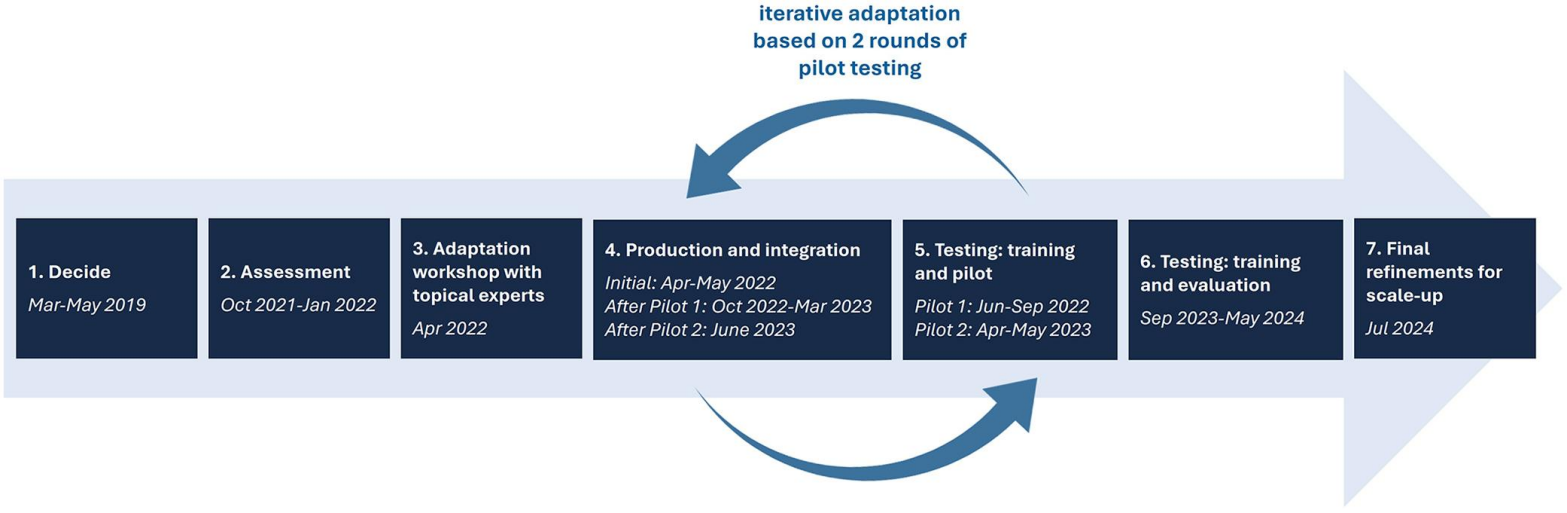
Kenya MOH ARCHES adaptation process.

The key adaptation steps for the Kenya MOH ARCHES adaptation are outlined below (Figure 1):

- **Step 1 – Decide:** In May 2019, The Kenya MOH decided to adapt and scale the Nairobi adaptation of the ARCHES model within the public-sector FP system nationally (starting in Uasin Gishu county) to help meet their national priorities to address and reduce GBV and unintended pregnancy. As the MOH had engaged with ARCHES and the study team as part of the Nairobi pilot, they were familiar with the model and supporting evidence from the Kenyan context. ARCHES was the only clinic-based model that had been recently tested within Kenya, engaged the MOH in the testing and dissemination of results, and addressed women's experiences with both IPV and RC thus leading to the MOH's decision to adopt the approach for public-sector scale-up. Other models were not considered.
- **Step 2 - Assessment:** A core tenant of ARCHES is to ground the intervention in the lived realities of women, providers, and the health system. To do this, detailed formative research was undertaken to inform adaptation. Semi-structured interviews (SSIs) and focus group discussions (FGDs) were conducted with key stakeholders to inform feasibility, acceptability, and scalability of the ARCHES approach; identify opportunities for adaptation within public health facilities in Uasin Gishu; and collect contextually relevant stories and quotes to feature in provider training materials. FGDs were conducted with IPV service providers/administrators (*n* = 1 FGD with 8 participants) and women and girls aged 15–49 seeking FP care at government health facilities (*n* = 4 FGDs with 6–8 participants each). SSIs were conducted with FP providers (*n* = 10 SSIs) and a subset of women/girls who reported experiencing RC or IPV in the past year (*n* = 10 SSIs). All SSIs and FGDs were audio recorded, transcribed verbatim by the interviewer, and translated to English. Transcripts were coded for common themes using a deductive approach to answer key adaptation questions (Supplementary Data Sheet 2) by two master's level research assistants in Dedoose. Key quotes were extracted to illustrate core themes.
  In addition to qualitative data, quantitative facility assessment checklists were also completed among all facilities targeted for implementation or testing (*n* = 24) to understand potential barriers and facilitators to ARCHES implementation at the facility level and systems preparation required prior to implementation. Data from paper checklists were entered in an Excel database and descriptive statistics summarized major findings. Quantitative and qualitative findings were triangulated by pre-determined adaptation questions in a matrix-based approach.
- **Step 3 - Adaptation workshop with topical experts**: A five-day adaptation workshop with national- and county-level MOH staff and ARCHES experts was held in Uasin Gishu county to review formative research findings and make high-level intervention and implementation adaptation decisions. A hierarchical adaptation decision tree (Supplementary Data Sheet 2) guided the workshop agenda in a linear process, as particular decisions informed others (for example, who would deliver ARCHES informed how training would proceed). The WHO ExpandNet, “Beginning with the End in Mind” guidance (52) was used to structure the workshop so participating stakeholders could consider modifications that would promote institutionalization and sustainability at county-level and national scale for each adaptation decision. Over 20 individuals attended the workshop including heads and managers from the Division of Reproductive, Maternal, Newborn, Child, and Adolescent Health; MOH Program Officers from FP and GBV programs; MOH monitoring and evaluation technical lead; Head of Uasin Gishu County Reproductive Health; Uasin Gishu County Reproductive Health Coordinators; champion providers and facility managers from Uasin Gishu; the Uasin Gishu county pharmacist; and partners from Population Council Kenya, University of California, and a private FP provider, all with prior experience implementing ARCHES in Nairobi.
  From the workshop, two teams were formed to guide adaptation and implementation. The first team, the *FP* *+* *Taskforce*, comprising of core project leads and decision-makers from the Kenya National MOH, Uasin Gishu County Department of Reproductive and Maternal Health, Population Council, and the University of California, made major decisions regarding adaptation, implementation, and institutionalization. The second team, the *FP* *+* *Implementation Team*, comprising of the local project coordinator and county implementing team (leadership and coordinators from the County Department of Reproductive and Maternal Health and provider-mentors) met regularly to carry out decisions of the FP + Taskforce and debrief on project implementation across facilities.
- **Step 4 – Production and integration**: Based on the decisions made during the adaptation workshop, national-level MOH staff integrated ARCHES elements into the standard FP counseling model adopted by the Kenya MOH and related training materials. This was done through both in-person and virtual meetings and independent work by staff members in regular weekly meetings. After this initial round of integration, two additional rounds of integration were needed ‒ one after each round of piloting ‒ to make refinements based on arising implementation challenges and pilot testing feedback.
- **Step 5 – Testing: training and pilot**: The adapted ARCHES model was pilot tested in two rounds in five health facilities. In the first round, 18 FP providers from three health facilities and six county-level supervisors were trained on the Kenya MOH's initial ARCHES adaptation, and in the second round, 22 FP providers from two new health facilities plus a subset of providers from the three original facilities were trained on a refined version of the model. Client exit interviews (round 1: *n* = 124, round 2: *n* = 120) were collected for one month during each pilot testing round to assess FP counseling quality, FP method selection, RC and IPV experience, and exposure to the ARCHES components. Additionally, during each pilot round, workshops (round 1: *n* = 5 with 12–24 participants, round 2: *n* = 1 with 25 participants) were held with the trained FP providers to get their feedback on their experience implementing the model. Key findings from both the client exit interviews and provider workshops were shared with the national-level MOH staff and further refinements to the ARCHES model were made after each round of pilot testing. While originally, only one pilot testing round was planned, poor intervention implementation in the first round of piloting led the project team to adopt major adaptations to intervention content and implementation strategies that required a second pilot.
- **Step 6 – Testing: training and evaluation:** The final version of the Kenya MOH ARCHES adaptation, designed for national scale-up in Kenya, was then implemented and evaluated in a hybrid implementation-effectiveness cluster randomized controlled trial (24 sites, *n* = 3809). Female FP clients aged 15–49 years at selected sites completed baseline surveys (immediately prior to receiving care), immediately post-visit exit surveys, and 6-month follow-up surveys. A detailed description of the full study protocol can be found elsewhere (22). Preliminary results indicate that the intervention improved contraceptive self-efficacy and reduced all forms of IPV as compared to controls. However, no gains were seen in contraceptive uptake, use, or incident pregnancy, except that clients who received the full intervention had improved contraceptive uptake at exit [forthcoming]. Spontaneous adaptations at the individual and personal level were documented through qualitative data collection with providers, clients, and provider-mentors.
- **Step 7 – Final refinements for scale-up:** Based on evaluation results on both effectiveness and implementation, and considerations for future scalability, the FP + Taskforce held a dissemination and scale-up workshop which shared results from the study, identified final refinements for the scalable model, and created county-level and national-level scale-up plans to guide next steps. Approximately 60 participants attended including the same representatives who were in the adaptation workshop with expanded reach to include all County Reproductive Health Coordinators involved in the project, champion providers and facility leads across the sub-counties, and leadership from several local non-governmental organizations who may be interested in implementing the approach in their own systems in the future.

### 2.3 Data sources

ARCHES modifications were documented in a variety of ways during the adaptation process. Adaptation decisions made during the five-day adaptation workshop and post-evaluation scale-up workshop (steps 3 and 6) were documented in final workshop reports. The adaptation workshop report guided the initial integration of the ARCHES elements into the MOH FP counseling model and training materials. During production and integration (step 4) and pilot testing (step 5), additional modifications were discussed and documented through notes taken during routine project team meetings. Routine meetings included monthly FP + Taskforce meetings and bi-monthly (i.e., every two weeks) FP + Implementation Team meetings. An adaptation tracker was used to document all proposed and final modifications throughout the adaptation process. It included information on proposed adaptations (description, date proposed, and person who proposed the adaptation), reason for the proposed adaptation, adaptation decisions (whether we decided to proceed with the proposed adaptation and why), and adaptation implementation information (date adaptation implemented and notes on implementation fidelity). The adaptation tracker was updated weekly by project staff during production and integration (step 4) and pilot testing (step 5). Adaptations documented in the adaptation workshop report and via routine meeting notes were incorporated into the tracker in addition to any other modifications identified and reported by project staff. Finally, post-implementation SSIs and FGDs with clients (*n* = 20 SSIs), providers (*n* = 10 SSIs, *n* = 2 FGDs), and provider-mentors (*n* = 2 FGDs) offered insights into spontaneous adaptations made during implementation (step 6) that informed final refinements into the model.

### 2.4 Adaptation characterization

**Figure 2:**
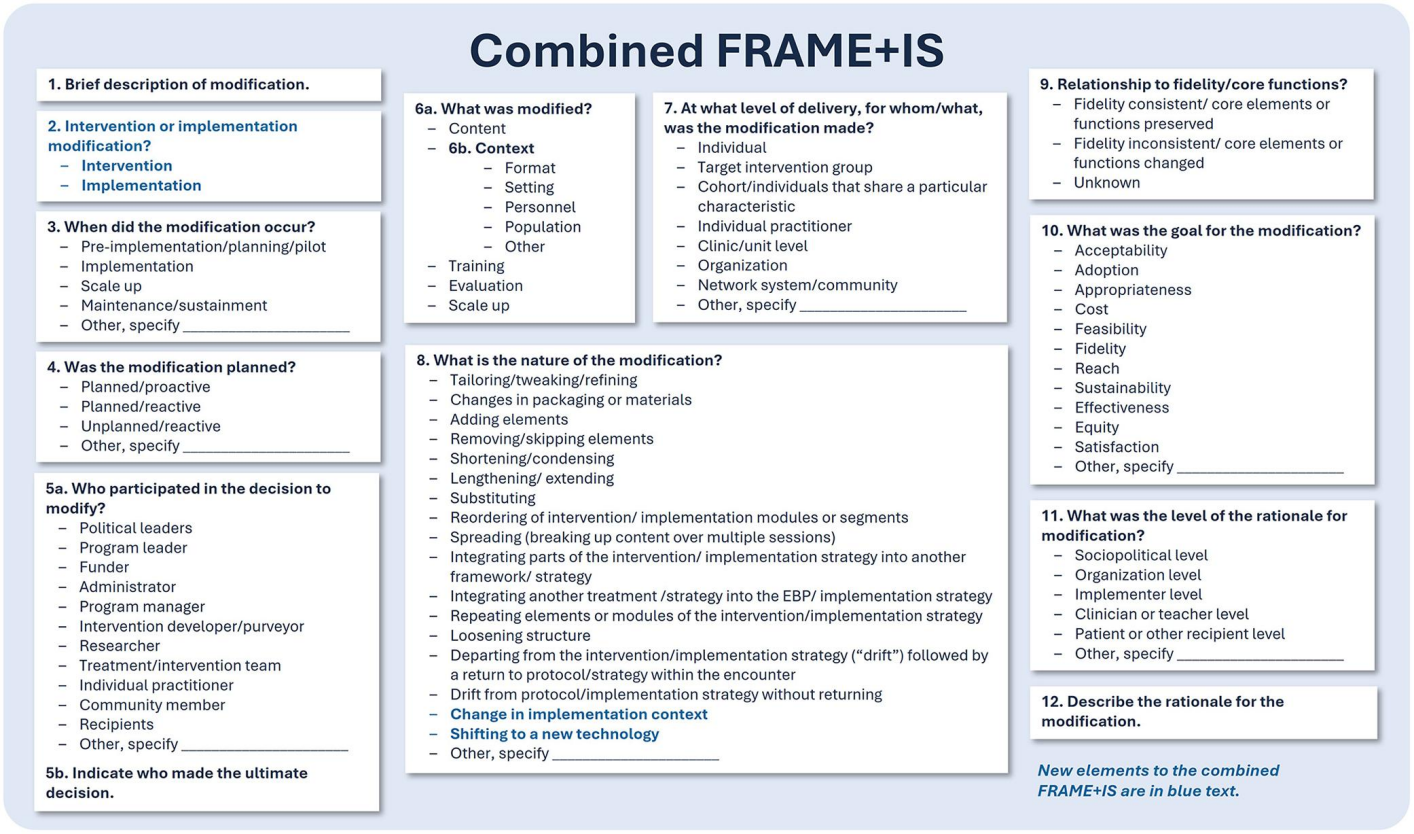
The combined FRAME + IS.

Two common adaptation-tracking frameworks, FRAME (44) and FRAME-IS (45), were adapted and retrospectively applied to systematically characterize the key modifications made to the ARCHES intervention and its implementation strategies. Recognizing overlap between FRAME and FRAME-IS, the two frameworks were combined into a single cohesive tool (FRAME + IS) to capture adaptations to both intervention content and implementation strategies. This integration reduced redundancy and enabled systematic and efficient tracking of modifications across both domains within one unified framework. The combined FRAME + IS includes the following 12 elements (Figure 2): 1) brief description of the modification, 2) whether the modification was to the intervention or implementation strategies, 3) when the modification occurred, 4) whether the modification was planned or unplanned, 5) who participated in the decision to modify, 6) what was modified, 7) at what level of delivery or for whom/what was the modification made, 8) the nature of the modification, 9) relationship to intervention fidelity/core functions, 10) goal of the modification, 11) level of rationale for the modification, and 12) description of the rationale for the modification. In combining the frameworks, all core elements of both FRAME and FRAME-IS were retained and one additional element was added – whether the modification was to the intervention content or implementation strategies (element 2). A few small additions to the categories were also made to better describe the nature of the modification (element 8) including “change in implementation context” and “shifting to a new technology”. Finally, based off the FRAME Excel tracking spreadsheet (53), a tracking table which includes all elements of the new combined FRAME + IS was created (Supplementary Table 1).

Two study team analysts, both masters-trained with eight or more years of post-graduation experience in program implementation and adaptation, from the University of California, led the adaptation tracking analysis. Their work was overseen by senior investigators and co-investigators with decades of experience in program adaptation and implementation in LMICs, and closely consulted with the MOH, implementers, and local study coordinator to ensure accuracy and transparency of results. Key ARCHES modifications were identified by reviewing the data sources described above and through discussions with project team members, particularly those closely involved in implementation such as the local study coordinator. Each key modification was then characterized across the 12 combined FRAME + IS elements by one analyst and then independently reviewed by the other analyst. Any characterization discrepancies were discussed and reconciled. Finally, the adaptation characterization was presented to the full study team for validation and refinement.

## 3 Results

### 3.1 Summary of adaptations

In adapting ARCHES for public sector facilities in Kenya, 12 key modifications were identified and characterized (Table 1). Modifications were evenly split between implementation strategy adaptations (50%) and intervention content adaptations (50%) with the majority being planned/proactive (75%). Most of the modifications occurred prior to implementation (83%) with the remaining occurring during implementation (8%) and scale up (8%). Over half were content modifications (58%) followed by contextual modifications (33%) and training modifications (8%). Almost all modifications were made at the network system/community level (92%) with one being made at the clinic/unit level (8%) which occurred during implementation. The nature of the modifications varied with the following being most prevalent: removing/skipping elements (33%), tailoring/tweaking/refining (25%), shifting to a new technology (17%), and shortening/condensing (17%). The most common reason for making modifications was feasibility (42%) followed by appropriateness (33%), sustainability (33%), acceptability (25%), effectiveness (25%), adoption (17%), fidelity (17%), and costs (8%). Overall, most adaptations were fidelity consistent (58%) with four modifications being fidelity inconsistent (33%) and one being unknown (8%) due to lack of detailed information on how it will be implemented. The Kenya MOH was the ultimate decisionmaker on all modifications taking into consideration input from ARCHES intervention experts, county-level MOH staff, FP providers, and FP clients.

**Table 1:** Summary of ARCHES adaptations (*n* = 12).

FRAME + IS Element	*n*	(%)
2. Type of modification		
Intervention	6	(50%)
Implementation	6	(50%)
3. When the modification occurred		
Initial institutionalization	1	(8%)
Pre-implementation/Planning/Pilot	9	(75%)
Implementation	1	(8%)
Scale up	1	(8%)
4. Whether the modification was planned		
Planned/Proactive adaption	9	(75%)
Unplanned/Reactive modification	3	(25%)
6. What was modified		
Content	7	(58%)
Contextual	4	(33%)
Training	1	(8%)
7. Level of delivery of the modification		
Network System/Community	11	(92%)
Individual	1	(8%)
8. Nature of the modification^a^		
Removing/skipping elements	4	(33%)
Tailoring/tweaking/refining	3	(25%)
Shifting to a new technology^b^	2	(17%)
Shortening/condensing (pacing/timing)	2	(17%)
Reordering of intervention/implementation modules	1	(8%)
Change in implementation context^b^	1	(8%)
Integrating parts of the intervention/implementation strategy into another framework/strategy	1	(8%)
9. Relationship to fidelity/core functions		
Consistent	7	(58%)
Inconsistent	4	(33%)
Unknown	1	(8%)
11. Rationale for modification^a^		
Feasibility	5	(42%)
Appropriateness	4	(33%)
Sustainability	4	(33%)
Acceptability	3	(25%)
Effectiveness	3	(25%)
Adoption	2	(17%)
Fidelity	2	(17%)
Costs	1	(8%)

a More than one category could be assigned to each modification.

b New element in the combined FRAME + IS.

### 3.2 Description of adaptations

#### 3.2.1 Intervention content adaptations

Shortly after the completion of the ARCHES Nairobi project (in 2020), the Kenya MOH **integrated content on RC and IPV into national FP guidelines** (adaptation 1, Table 2). This was done rapidly to take advantage of a rare policy window: the national FP guidelines were undergoing revision, an opportunity that only arises every five years. These adaptations marked a significant step toward embedding RC and IPV within national policies, following the promising results of the ARCHES Nairobi trial. However, given the rapid timeline, there was limited time to engage with ARCHES implementers or researchers and refine materials during the initial integration leading to several misalignments with the Nairobi model. For example, the guidelines included the definition of RC and IPV and their connection to women's family planning outcomes but omitted key ARCHES components including screening, response, and referral strategies. While these changes aimed to raise awareness, particularly of RC, they did not fully equip providers to address RC and IPV in practice. In the subsequent adaptation phase, however, the MOH engaged in a collaborative process with ARCHES experts to revise and strengthen these materials. This helped align the national guidelines with many of the intervention's core components, which were ultimately updated in the final national materials during the next guideline review window. This collaboration represented meaningful progress toward institutionalizing a more comprehensive approach to addressing RC and IPV within Kenya's public-sector FP services.

**Table 2:** Key adaptations of the ARCHES model for implementation at scale in Kenya using the combined FRAME + IS.

No.	1. Modification description	3. When?	4. Planned?	6. What was modified?	7. At what level of delivery?	8. Nature of modification	9. Fidelity-consistent	11. Rationale for modification?
Intervention content modifications								
1	Integration of RC/IPV content into national FP guidelines	Initial institutionalization	Yes	Content	System/Community	Integrating parts of the intervention into another strategy	No	Adoption
2	Changing the order in which the ARCHES components were implemented	Pre-implementation/Planning/Pilot	Yes	Content	System/Community	Reordering of intervention modules or segments	Yes	Feasibility
3	Discreet method use information not formally institutionalized in written materials	Pre-implementation/Planning/Pilot	Yes	Content	System/Community	Removing elements	No	Appropriateness
4	Adjusting the RC screening & counseling language used with clients	Pre-implementation/Planning/Pilot	Yes	Content	System/Community	Tailoring/refining	Yes	Effectiveness Appropriateness Feasibility Acceptability
5	Adjusting the IPV screening & counseling language used with clients	Pre-implementation/Planning/Pilot	Yes	Content	System/Community	Tailoring/refining	Yes	Effectiveness Appropriateness Feasibility Acceptability
6	Deviations in the flow of the counseling algorithm based on individual client characteristics	Implementation	No	Content	Individual	Removing elements	No	Appropriateness
Implementation strategy modifications								
7	Implementation in the public sector	Pre-implementation/Planning/Pilot	Yes	Contextual (setting)	System/Community	Change in implementation context^a^	Yes	Adoption
8	In-person IPV referrals to trained medical social workers within the facility	Pre-implementation/Planning/Pilot	Yes	Content	System/Community	Tailoring/refining	Yes	Effectiveness Feasibility
9	Shortening the ARCHES provider training and integrating it into the Kenya MOH six-day comprehensive FP training	Pre-implementation/Planning/Pilot	Yes	Training	System/Community	Shortening + Removing elements	No	Sustainability
10	Provider mentorship modality and schedule	Pre-implementation/Planning/Pilot	Yes	Contextual (personnel)	System/Community	Shortening + Removing elements	Yes	Fidelity Sustainability Feasibility
11	Using the Kenya FP + mobile application	Pre-implementation/Planning/Pilot	No	Contextual (format)	System/Community	Shifting to a new technology^a^	Yes	Fidelity Acceptability Sustainability
12	Digitizing the mini-booklet	Scale up	No	Contextual (format)	System/Community	Shifting to a new technology^a^	Unknown	Costs Sustainability

a New element in the combined FRAME + IS.

For the Kenya MOH adaptation, ARCHES elements were integrated into the Balanced Counseling Strategy Plus (BCS+) FP counseling protocol, the government-approved standard of FP counseling care in Kenya. BCS + is a client-centered, interactive counseling approach designed to improve quality of care and support clients to make informed decisions about contraceptive use (54). BCS + also integrates screening for other health issues (HIV, cervical cancer, etc.) after contraceptive counseling and selection. ARCHES elements were integrated into both the BCS + algorithm and counseling cards (see Supplementary Data Sheets 3, 4). The **order in which the ARCHES elements were implemented changed** (adaptation 2, Table 2) to allow for alignment with the BCS + counseling algorithm: first counseling and screening for RC; then, separately after selecting a contraceptive method, counseling and screening for IPV; and finally offer of an educational mini-booklet on RC, IPV, contraception, and referral services. Information on discreet use, while offered first in the Nairobi ARCHES model, was offered after RC screening for those who disclosed RC.

**Information on discreet contraceptive method use was not formally institutionalized in written materials including provider job aids and training presentations** (adaptation 3, Table 2) due to the MOH's concerns that this could provoke backlash and their desire to formally align with existing policies that encourage male partner involvement in family planning. Instead, the following more general statement was included in the counseling algorithm for clients who screened positive for RC: “*Discuss strategies to use the family planning method of their choice that is most convenient or works best for them*”. Information on discreet use was shared verbally with providers during training through interactive discussions (prompt: “*What are some of the strategies that a client facing RC can employ in order to use her chosen FP method successfully?*”) and role plays which featured clients who were experiencing RC. Providers were also strongly encouraged to counsel all clients on discreet method use strategies during mentorship visits.

**Adjustments were made to the RC and IPV counseling and screening language** (adaptations 4 and 5, Table 2) to improve client understanding, conform with provider counseling preferences, and better align with Kenya MOH guidelines. The counseling content was adjusted to include contextually relevant examples of RC and explanations of the health consequences of both RC and IPV to increase client awareness. RC and IPV screening questions were consolidated from multiple items on each form of abuse (i.e., RC - pregnancy coercion, contraceptive sabotage; IPV - physical, sexual, and emotional abuse) into a single question for each. Revised questions included context on the prevalence and forms of RC and IPV, explained why providers ask about them, and invited clients to share if they faced similar challenges. These changes were made based on provider feedback that the Nairobi ARCHES model questions felt repetitive and impersonal and that a conversational, narrative approach was more engaging than a checklist format.

During implementation, **some providers reported deviating from the flow of the counseling algorithm and skipping content based on individual client characteristics** (adaptation 6, Table 2). For example, some providers reported skipping the IPV section of counseling for single mothers because they assumed single women did not need this counseling. Providers reported adjusting the flow and content of counseling on a case-by-case basis to meet their perceived needs of the client. These deviations, however, ran counter to the core ARCHES value to offer universal counseling despite disclosure or perceived risk.

#### 3.2.2 Implementation strategy adaptations

For this Kenya MOH ARCHES adaptation, **implementation shifted to public sector health facilities** (adaptation 7, Table 2) in Uasin Gishu county which was selected as the first county in Kenya to test the adapted ARCHES approach. In Kenya, health services are decentralized from the national government system, and counties are responsible for implementation in accordance with national guidelines, training, and materials. Given this contextual shift, Uasin Gishu county health services staff were responsible for implementation of the adapted ARCHES model while national-level MOH staff made all final adaptation decisions, led initial provider trainings, and drafted materials with county-level personnel providing input throughout the process.

**For clients who screened positive for IPV, warm referrals were primarily done in person by connecting the client with a trained medical social worker within the facility** (adaptation 8, Table 2) instead of over the phone. In Uasin Gishu county, there are not many in-person IPV support centers available, and it would be difficult for many clients to access them due to long travel distances. By providing IPV training for the medical social workers already available in most health facilities, the intervention took advantage of an already existing referral resource to make it easier for women to access IPV support services.

To promote sustainability of the approach, **the ARCHES provider training was shortened to three hours and integrated into the standard Kenya MOH six-day comprehensive FP training** (adaptation 9, Table 2). The amount of time spent on the ARCHES content was drastically reduced to allow for sufficient time to train on other family planning elements including counseling, method-specific information, and method delivery/insertion. In the Nairobi ARCHES model, three full days were dedicated solely to ARCHES content and practice. In contrast, the MOH revision allocated approximately three hours to ARCHES content. This included educational sessions followed by counseling role plays during which providers were asked to practice both the ARCHES content and the other family planning counseling content they had learned.

To reinforce and build upon training content, post-training mentorship is a necessary extension of ARCHES training which helps build providers' confidence and practical skills and supports fidelity to the ARCHES model. **The FP provider mentorship modality and schedule was adjusted for implementation in Uasin Gishu county** (adaptation 10, Table 2). In the Nairobi adaptation, mentorship was conducted as often as needed, even daily if required. For the MOH adaptation, mentorship visits were conducted by FP providers, who attended a half-day mentorship orientation, on the following schedule: weekly visits the first month post-training (four visits), bi-weekly visits the second month post-training (2 visits), and monthly visits the third month post-training (one visit). Following the third month, mentorship was standardized within regular external supervision visits, occurring approximately once per quarter. Formalizing the mentorship schedule, rather than having mentorship on an as-needed basis, decreased the number of mentorship sessions required, allowing for a more sustainable model for future expansion and scale-up. This also increased the feasibility of planning and budgeting for mentorship.

During the first round of pilot testing, FP providers found using the paper-based BCS + algorithm and counselling cards with integrated ARCHES content cumbersome due to its complexity compared to standard screening protocols. Even though BCS + was endorsed by the Kenya MOH and included in intermittent FP trainings, its implementation was not uniformly applied by all FP providers nation-wide. Thus, most providers in Uasin Gishu County involved in the project were unfamiliar with the use of BCS + prior to the pilot training. Many reverted to their previous method of FP counseling, GATHER, after training and, therefore, did not implement the adapted ARCHES strategies with fidelity. Based on poor rates of implementation of ARCHES in client exit surveys and qualitative feedback from providers and provider-mentors about the challenges in implementing BCS + counseling using the paper-based tools, the MOH decided to develop and introduce a **mobile application to guide providers through the BCS** **+** **with integrated ARCHES elements** (adaptation 11, Table 2) in a step-by-step process to improve fidelity to the model and reduce the provider decision-making burden and time needed for counseling. The app was loaded onto providers' personal smartphones and was thereby a more sustainable mechanism than paper-based materials which often went missing and were expensive to print and distribute. Preliminary findings from the hybrid implementation-effectiveness trial show that use of the app increased client quality of care and ARCHES fidelity [forthcoming]. The mobile application, **Kenya Family Planning Plus** (Kenya FP+), is available on the Google Play store for Kenya-based providers. It is free to download and works offline.

The last implementation strategy adaptation was documented during the final scale-up workshop. The Kenya MOH would like to **digitize the ARCHES educational mini-booklet** (adaptation 12, Table 2; Supplementary Data Sheet 5) in the future, so clients can access it on their smartphones. This shift to a digital version of the booklet would promote sustainability and lower intervention costs but is yet to be implemented or evaluated in Kenya.

## 4 Discussion

This study provides new insights into how collaborative, government-led adaptation processes can support the institutionalization of EBIs in addressing RC and IPV within public sector FP services. Twelve key modifications were identified and characterized using FRAME + IS, a new unified tool designed to capture both intervention and implementation strategy adaptations. Unlike many documented adaptation efforts (55–57) – which often rely on reactive, mid-implementation modifications in response to emergent challenges – the majority of changes in this study were made proactively during planning and pilot testing in our adaptive management approach which prioritized continual learning and refinements. Importantly, this work was embedded within a hybrid implementation-effectiveness trial which, because of ongoing data collection with clients and focus on implementation measurement, allowed for responsiveness to real-world conditions. The adaptive management approach intentionally accommodated both planned and emergent adaptations, recognizing that successful implementation in dynamic public sector settings requires iterative refinements. Rather than viewing these as deviations from protocol, they reflect the reality of conducting pragmatic implementation research in service delivery contexts and are central to understanding how interventions function under routine conditions. Notably, this adaptation took a Cadillac approach and future efforts, with minimal time and resources, may require a pared down, participatory adaptation approach, as has been conducted in other settings, like India [forthcoming].

Several strategic adaptations were instrumental to promoting feasibility, fidelity, and sustainability of the ARCHES model. Integration into national tools enabled institutionalization at scale but also introduced challenges. BCS + is a structured, integrated, and time-intensive model, which is endorsed by the Kenya MOH, but has yet to be successfully implemented nation-wide; most providers were unfamiliar with it, and despite substantial training and mentorship, some remained resistant, citing its complexity and poor fit with their workflow. This misalignment between policy-level standardization and provider and clinic realities of high-volume service delivery reflects findings from prior studies emphasizing the need to align intervention protocols with provider capacity and clinic context (58, 59). It also underscores the broader challenge of integrating new components into an overstretched health system, where staffing shortages, supply disruptions, and competing demands are routine. Successful integration required not just technical adaptation but alignment with existing service delivery infrastructure. To further reduce complexity, paper-based materials were replaced with a mobile application (Kenya FP+), which guided providers through the counseling algorithm in an intuitive, accessible format. This digital solution improved fidelity, quality-of-care, and reduced provider burden [forthcoming], echoing evidence from similar settings (60, 61); digital tools should be further considered in scale-up efforts where feasible. Finally, mentorship, critical to providers' learning, was adapted into a formalized, time-bound model, making it more feasible to implement and budget for at scale. Together, these adaptations reflect how real-world constraints shape decisions around balancing complexity with fidelity, feasibility, and sustainability. To support effective roll-out in Kenya, however, continued investment will be needed to secure stable funding, streamline in-service training and mentorship, and incorporate ARCHES, BCS+, and RC/IPV content into pre-service provider education. It is notable that to be implemented effectively the ARCHES model had to be expanded in job aids, training, and mentorship time compared to the U.S. approach which will likely have implications on sustainability.

A distinctive and novel feature of this project was the Kenya MOH's leadership and ownership of the entire adaptation process. While government-led adaptation has been explored in some HIV and maternal health efforts (48, 49), this study represents a novel contribution in the context of reproductive agency and GBV response. Unlike many scale-up efforts led by donors or implementing partners, the MOH drove all major decisions demonstrating a rare level of political will to institutionalize an intervention addressing RC and IPV within national family planning services. This leadership was critical to aligning the intervention with broader government priorities, including efforts to reduce unintended pregnancy and address GBV through the public health system. MOH engagement accelerated uptake and integration into national policies, training materials, and data systems, and positioned ARCHES for long-term sustainability. However, the MOH-led adaptation did not immediately translate into sustainable domestic financing for continued implementation and scale. As in other settings, high-level policy support does not always guarantee financial investment, particularly in resource-constrained health systems (62). At the time of writing, further expansion remains reliant on external donor support, with no earmarked budget line for ARCHES or related RC/IPV activities within national or county-level financing structures, despite post-implementation advocacy efforts advocating for this allocation. This gap underscores the need for intentional planning around financial sustainability from the outset—including integration into national costed implementation plans, budgeting cycles, and domestic resource mobilization strategies ideally in collaboration with funding or multi-lateral support organizations.

Early fidelity risks also emerged during the MOH-led integration. For instance, initial registers and KHMIS reporting combined RC and IPV into a single construct and lacked clear guidance on screening, response, and referral departures from core ARCHES components. These challenges reflect tensions documented in other government-led scale-up efforts, where strong local ownership can drive sustainability but may dilute intervention fidelity without structured technical support (30, 31). In this case, ongoing collaboration between the MOH and ARCHES experts helped resolve many early misalignments. However, some challenges persisted due to political and gendered constraints, which likely affected implementation fidelity. For example, some core ARCHES elements, such as universal discreet contraceptive use counseling, were not institutionalized, illustrating the compromises often made when aligning evidence-based models with national policy and health system priorities which may deemphasize female autonomy. This experience highlights how meaningful systems change often requires navigating the intersection of political will, technical integrity, and contextual realities. In practice, not all evidence-based elements can be implemented exactly as designed; at times, certain effective components must give way to broader system priorities, feasibility concerns, or political sensitivities.

To systematically track adaptations, we developed **FRAME** **+** **IS**, a combined tool that integrates the FRAME and its implementation strategy counterpart, FRAME-IS. While each framework has been applied separately across a range of global health and behavioral interventions (44, 45, 63), this is the first published example of a unified framework applied to track adaptations across both domains concurrently. Combining the two reduced redundancy and allowed for more efficient, holistic documentation, especially in complex system-level adaptations. For other governments or implementing partners considering similar adaptations, this project offers a practical model for how structured collaboration and shared decision-making can enable context-responsive changes without losing sight of core intervention components. The FRAME + IS tool provides a replicable mechanism for systematically documenting and assessing adaptations, which is especially important for government-led initiatives where modifications are inevitable due to political, logistical, or cultural factors. Its application here advances the field by offering a concrete, easy-to-use resource for adaptation tracking in global health programming. We recommend that future initiatives seeking to adapt for scale consider applying this approach in real time, rather than retrospectively, to build transparency and alignment between MOH actors and technical partners.

### 4.1 Limitations

This study has several limitations. First, although we systematically tracked adaptations using workshop reports, meeting notes, and an adaptation tracker, it is possible that spontaneous or informal adaptations, especially at the provider or facility level, were missed. Real-time observation, rapid ethnographic methods, or multiple rounds of adaptation-focused data collection may be better suited for capturing these dynamic, context-specific changes, though they require greater time and financial investment (63–65). Second, this work was conducted within the context of a hybrid effectiveness-implementation trial, which included intensive mentorship, ongoing data collection, and close collaboration with government stakeholders. These enhanced support structures may not reflect typical implementation conditions, and additional adaptations are likely to emerge as the model is scaled across counties in Kenya. As the model is rolled out in Kenya, it is nearly certain that other adaptations will take place which are not recorded here. Additionally, the adaptation on a digitized client education mini-booklet was determined but not implemented prior to this studies publication, thus the authors are unable to report on its utility and acceptability in practice.

## 5 Conclusion

This study represents the first known example of a government institutionalizing an intervention to address RC and IPV within public sector family planning services. Through an early, proactive, and collaborative adaptation process, the ARCHES model was modified for scale while retaining many of its core components. This work offers a rare example of RC and IPV integration into routine public health services, led by a government actor, and highlights both the potential and trade-offs of such an approach.

Additionally, the development and application of FRAME + IS adds a practical and replicable tool for future adaptation efforts particularly within complex systems. As global health systems continue to scale interventions for sexual and reproductive health and rights and GBV, structured adaptation processes and tools like FRAME + IS will be critical to balancing fidelity and fit for sustainable impact.

## Data Availability

The original contributions presented in the study are included in the article/Supplementary Material, further inquiries can be directed to the corresponding author.

## Footnotes

1 Silverman J, Uysal J, Undie CC, Liambila W, McDougal L, Pearson E, et al. Effects of a clinic-based reproductive empowerment intervention on reproductive coercion, intimate partner violence, and pregnancy outcomes: A cluster-controlled trial in Nairobi Kenya. (under review).
