# Supplementary material for: Strengthening Kenya's public health response to reproductive coercion and intimate partner violence in family planning clinics: applying the FRAME + IS approach
Source: Front Reprod Health. 2026 Jan 5;7:1630877. doi: 10.3389/frph.2025.1630877 (PMC12813199; doi:10.3389/frph.2025.1630877)
Supplement: Supplementary file 2 [file Datasheet1.pdf]

MY FP CHOICE • MY RIGHT

GATTHER

- **G** — Greet the client and assure confidentlaity.
- **A** — Ask the client about her FP needs.
- **T** — Tell her about FP methods including types and ways to use methods privately.
- **T** — Talk to her about RC and IPV.
- **H** — Help her select a method.
- **E** — Explain how to use her preferred method and ways to use the method privately.
- **R** — Referral (warm), mini-booklet, and offering male engagement.

Conduct the ARCHES intervention with all family planning clients but only when you can speak to her alone.

GREET THE CLIENT and ASSURE CONFIDENTALITY

SCRIPT: *"I'm really glad you came in today. Before we get started, I want you to know that everything you share with me will not leave this room. I will not tell anyone; not your family, partner, or friends, unless you tell me to do so."*

TELL HER ABOUT FAMILY PLANNING METHODS THAT CAN BE USED PRIVATELY, WITHOUT ANYONE KNOWING

SCRIPT: *"We have found that many women in Kenya have difficulty using FP due to their partners or family. To help our clients make an informed choice we provide information about how to use FP methods without anyone knowing to all of our clients."*

Coil [IUCD]	Very Effective	Easy to use privately	We can cut IUD strings short to 2-3 cm immediately after insertion so your partner will not feel it.
Injection	Very Effective	Easy to use privately	You can come to the clinic for the injection when your partner is away.
Implant	Very Effective	Possible to use privately	You can wear long sleeves or another garment that covers the injection site until any bruising or swelling has gone down. You can tell your partner that the scar is from a boil or an abscess that was removed by your provider.
Contraceptive Pills	Effective if taken daily	Possible to use privately	You can hide the pills in a place your partner would not look (e.g. friend's house, office, coal bag, another medication bottle) and take the pills while he is out of the house.
Male/female condoms,, standard days, withdrawal	Somewhat effective	N/A	These methods will require the cooperation of a male partner.
LAM, Sterilization, EC	Very effective	Varies	Only counsel on these methods if she has had her baby within the last 6 months, does not want to have anymore children, or has had unprotected sex in the last 120 hours. Refer to flip book for counseling information on partner detection.

TALK TO HER ABOUT REPRODUCTIVE COERCION

SCRIPT: "Unfortunately, many women have a difficult time using family planning with their partners, so we ask these questions of all our clients."

- Have you ever felt pressured or forced by your current partner to become pregnant when you did not want to be?
- Has your current partner ever made it difficult for you to get family planning or to use family planning? (e.g. destroy, take away, or hide your contraception)

RC PRESENT	RC NOT PRESENT
SCRIPT: "Thank you for sharing this with me, I know these situations can be difficult to talk about. You have the right to be in control of your reproductive choices, no matter your situation. We can help and give you information and strategies to help you make the right FP decisions for yourself."	SCRIPT: "I am glad to hear that you are not experiencing any of these issues in your relationship today. If your situation ever changes, I want to assure you that you have a safe space here to talk about these issues and receive help."

TALK TO HER ABOUT IPV

SCRIPT: "Because so many of the women and girls that we talk to are treated badly by their partners and husbands, and we can connect them to helpful services we talk about these experiences with all of our clients."

- Are you currently in a relationship with a partner who physically hurts you?
- Are you currently in a relationship with a partner who threatens, frightens, insults you, or treats you badly?
- Are you currently in a relationship with a partner who forces you to have sex or to do something sexual that makes you feel uncomfortable?

IPV PRESENT	IPV NOT PRESENT
SCRIPT: "This must be a difficult situation. Thank you for sharing this with me. You have the right to be in a safe and non-violent relationship and we can connect you to services that can help."	SCRIPT: "I am glad to hear that you are not experiencing any of these issues in your relationship today. If your situation ever changes, I want to assure you that you have a safe space here to talk about these issues and that we can connect you to services that help women who are experiencing violence."

OFFER WARM REFERRAL IF IPV POSITIVE

SCRIPT: *"My colleague [NAME], who works at [MSF/GBVRC], has helped many of my clients who are experiencing abuse from a partner. Would you be interested in speaking with her?"*

CLINIC	PROVIDER CONTACT	GENERAL PHONE
Gender Based Violence Resource Center (GBVRC)	Getter Wasilwa, Psychologist, 0722-882932	0202726300/ 2726300 ext. 43136
Médecins Sans Frontières (MSF) Lavender Clinic	Mercy Githara, Clinical Psychologist, 0720-890258	0711400506

OFFER MINI-BOOKLET FOR ALL CLIENTS

SCRIPT: *" Many women find the information in this booklet helpful, for both themselves and to share with other women they know. Not everyone feels safe to take this with them, so you can also read it here and leave it, or come back another time to read it."*
