# Supplementary figures and images for: Strengthening Kenya's public health response to reproductive coercion and intimate partner violence in family planning clinics: applying the FRAME + IS approach
Source: Front Reprod Health. 2026 Jan 5;7:1630877. doi: 10.3389/frph.2025.1630877 (PMC12813199; doi:10.3389/frph.2025.1630877)

# ARCHES

## Adaptation

### Decision Tree

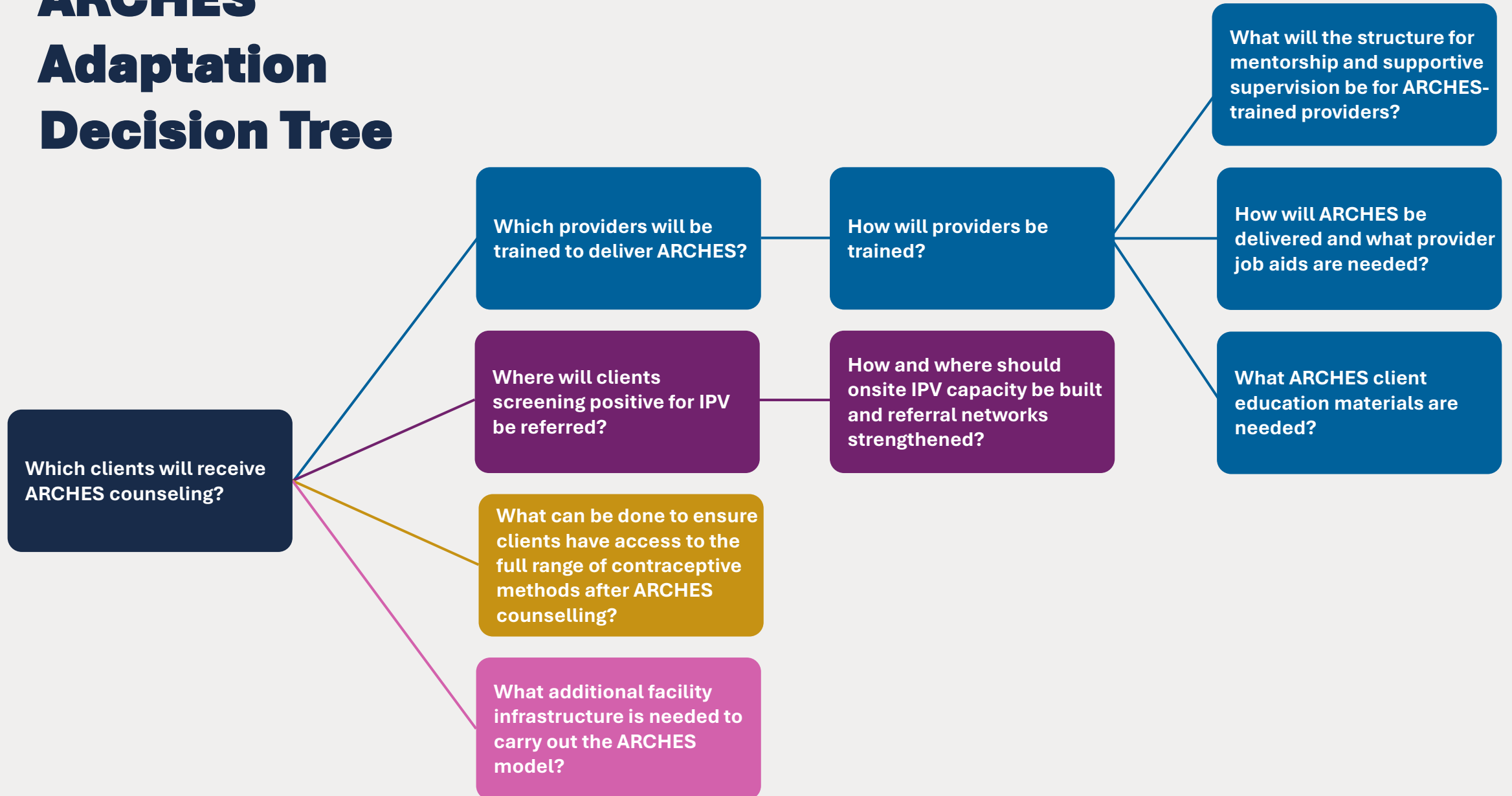

Supplement: Supplementary file 3 [file Datasheet2.pdf]
