# Supplementary material for: Strengthening Kenya's public health response to reproductive coercion and intimate partner violence in family planning clinics: applying the FRAME + IS approach
Source: Front Reprod Health. 2026 Jan 5;7:1630877. doi: 10.3389/frph.2025.1630877 (PMC12813199; doi:10.3389/frph.2025.1630877)
Supplement: Supplementary file 4 [file Datasheet3.pdf]

REPUBLIC OF KENYA

Ministry of Health

ALGORITHM FOR USING THE BALANCED COUNSELING STRATEGY PLUS (BCS+)

PRE-CHOICE STAGE

1. Establish and maintain a warm, cordial relationship.
2. Inform client (and partner, if present) that there will be opportunities to address both health and family planning needs during this consultation.
3. Ask client about current family size and current contraceptive practices. Counsel the client using the "**Healthy Timing and Spacing of Pregnancy"** counseling card.
4. If client is currently using a family planning method or delaying pregnancy, ask about her/his satisfaction with it and interest in continuing or changing the method.
5. If partner is present, use the "**Male Services and Support**" counseling card.
6. Rule out pregnancy by using the checklist on the "**How to be Reasonably Sure a client is not pregnant" Card.**
7. Display all of the method cards. Ask client if she/he wants a particular method. Ask all of the following questions by setting aside method cards based on the client's responses:
   a) Do you wish to have children in the future? If "**Yes,**" set aside "**Vasectomy**" and "**Bilateral Tubal Ligation**" cards. Explain Why. If "**No**," keep all cards and continue.
   b) Have you given birth in the last 48 hours? If "**Yes**," set aside "**Combined oral contraceptives**" (the Pill) and "**Combined injectable Contraceptive**", "**Combined vaginal ring**" and "**Combined hormonal Skin Patch**" cards. Explain why. If **"No,"** continue with the next question.
   c) Are you breastfeeding an infant of less than 6 months old? If "**Yes**," set aside the "**Combined oral contraceptives**" (the Pill), "**Combined injectable Contraceptive**", "**Combined vaginal ring**" and "**Combined hormonal skin Patch**" cards. Explain why. If "**No**," or she has begun her monthly bleeding again, set aside the **"Lactational Amenorrhea Method (LAM)"** card. Explain why.
   d) Do you have any medical conditions? Are you taking any medications? If "**Yes**," ask further about which conditions or medications. Refer to **Medical Eligibility Criteria Wheel** and set aside all contraindicated method cards. Explain why. If "**No**," keep all the cards and continue.
   e) Are there any methods that you do not want to use or have not tolerated in the past? If "**Yes**," **probe** further and set aside the corresponding method cards which the client is not comfortable using or has not tolerated in the past. If "**No**," keep the rest of the cards.
   f) Does your partner support you in family planning? If "**YES**", continue with the next question. If "**NO**", set aside the following cards: "**Vasectomy**", "**female condom**", "**male condom**", "**all Natural FP methods**" and "**withdrawal**". Explain why.

METHOD CHOICE STAGE

8. Arrange the cards that have not been set aside in order of their effectiveness (from highly effective to less effective) and briefly review the attributes of each method.
9. Use the "**Reproductive Coercion Counselling (RC)"** card to provide client with information on RC and screen for RC.
10. Ask the client to choose the method that is most convenient for her/him.
11. Using the **MEC wheel**, check whether the client has any medical condition which makes her/him not eligible for any method:
    a) Review "Method not advised using **Medical Eligibility Criteria**" and explain to the client why she/he cannot use the method.
    b) Ask the client to select another method from the remaining cards and use the **MEC wheel** to verify if the client is eligible for the method chosen.

POST-CHOICE STAGE

12. Discuss the method chosen with the client using the **BCS + Cards** as a counseling tool. Determine the client's comprehension and reinforce key information.
13. Make sure the client has made a definite decision. Give her/him the method chosen, referral if need be, a back-up method depending on the method selected, and a return date.
14. Talk about the benefits of involving the partner in decisions about use of contraception.

SYSTEMATIC SCREENING FOR OTHER HEALTH SERVICES STAGE

15. Using information collected previously, determine client's need for post-pregnancy, breastfeeding, newborn, infant care, well-child services or post abortion care.
    a) If the client reported giving birth recently, review the "**Promoting a Healthy Postpartum Period for the Mother**" and "**Promoting Breast Feeding, Newborn and Infant Health**" cards with client. Provide or refer for services, if need be.
    b) For clients with children less than 5 years of age, ask if children have been taken to well-child services. Provide or refer for immunizations and growth monitoring services, if need be.
    c) If the client reports a recent abortion, review the" **Post Abortion Care"** card. Provide or refer for post abortion care services, if need be.
16. Discuss with the client STI/HIV transmission and prevention and dual protection using the relevant counselling cards.
17. Conduct STI and HIV risk assessment using the relevant counseling card. If symptoms are identified, provide treatment.
18. Ask the client whether she/he knows her/his HIV status.
    a) If the client knows that she/he is HIV positive:
       - Review the "**Positive Health, Dignity and Prevention"** counseling card
       - Refer the client to center for wellness care and treatment.
    b) If the client knows that she/he is HIV negative:
       - Discuss a time frame for repeat testing
       - Review the "**Dual Protection and STI/HIV Transmission and Prevention"** card.
    c) If the client does not know her/his HIV status:
       - Discuss HIV Counseling and Testing (HCT) with client using "**HIV Counseling and Testing"** card
       - Offer or initiate testing with client, according to national HIV Protocols
       - Counsel the client on test results:
         - If the client is HIV positive, review "**Positive Health, Dignity and Prevention**" counseling card and refer client to center for wellness care and treatment
         - If the client is HIV Negative, counsel using "**Dual Protection and STI/ HIV Transmission and Prevention**" card
         - Discuss time frame for retesting for HIV
         - Offer condoms and instructions on correct and consistent use.
19. Ask the client if she has been screened for Tuberculosis (TB) in any previous FP visit:
    a) If not, screen for TB using the "**Screening for Tuberculosis"** card.
    b) If negative, encourage the client to undergo screening again at her next visit.
    c) If TB symptoms are identified, refer for treatment.
20. Ask the client when she had her last screening for Cervical Cancer (HPV or VIA or Pap smear) or Breast Cancer.
    a) If her last Cervical Cancer screening was done more than 5 years ago, counsel her on the importance of screening today using the "**Screening for Cervical Cancer** "card.
    b) If she is a known HIV positive client and screening was done more than 1 year ago, counsel her on the importance of screening today using the "**Screening for Cervical Cancer"** card.
    c) Provide or refer for services.
    d) If her last Cervical Cancer screening was less than 5 years ago continue with next question.
    e) Review the "**Breast Cancer Information and Awareness**" card with the client.
21. Counsel the client using the "**Intimate Partner Violence (IPV) Screening and Support**" card. If client shows any signs of IPV refer her for specialized services.
22. Give follow-up instructions and the "**Brochure**" for the method chosen and "**My family Planning Choices and Rights**" booklet. Set a date for next visit to the health facility.
23. Thank her/him for the visit and complete the counseling session.

Copyright © 2023, Ministry of Health, Government of Kenya.
Any part of this document may be freely quoted or reproduced, provided the source is acknowledged.
It may not be sold or used for commercial purposes or for profit.
For more information and additional copies contact"
The Division of Reproductive and Maternal Health, Ministry of Health
P.0. Box 43319 GPO 00100 Nairobi, Kenya www.familyhealth.go.ke
Email:headrmhke.moh@gmail.com

World Health Organization

Division of Reproductive and Maternal Health

Every life counts
