# Supplementary material for: Strengthening Kenya's public health response to reproductive coercion and intimate partner violence in family planning clinics: applying the FRAME + IS approach
Source: Front Reprod Health. 2026 Jan 5;7:1630877. doi: 10.3389/frph.2025.1630877 (PMC12813199; doi:10.3389/frph.2025.1630877)
Supplement: Supplementary file 5 [file Datasheet4.pdf]

MINISTRY OF HEALTH

REPRODUCTIVE COERCION COUNSELLING

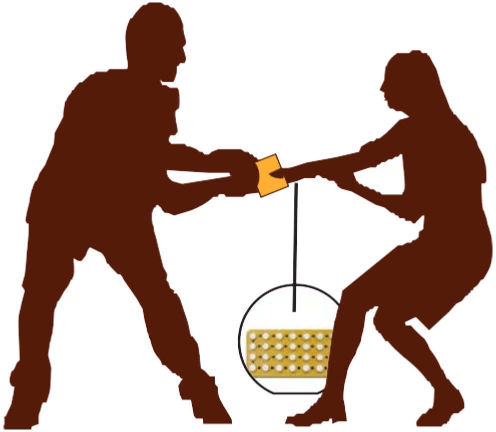

FAMILY PLANNING CHOICES

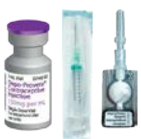

INJECTION

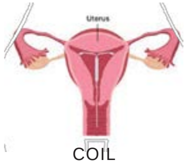

COIL

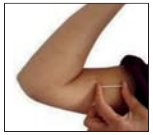

IMPLANT

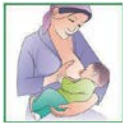

LAM

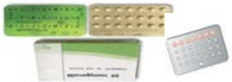

PILLS

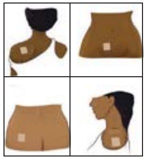

SKIN PATCH

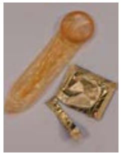

CONDOMS

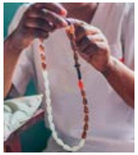

STANDARD DAYS METHOD

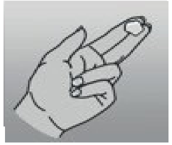

TWO DAYS METHOD

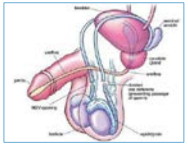

VASECTOMY

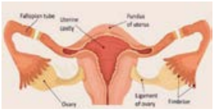

LUBAL LIGATION

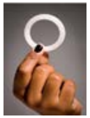

VAGINAL RING

MINISTRY OF HEALTH

REPRODUCTIVE COERCION COUNSELLING

Assess the client's risk for reproductive coercion (RC). Provide information to the client to help her make informed decisions in relation to FP use.

1. Share information on reproductive coercion with the client:
   - Some clients have male partners or family members who make it difficult to use family planning.
   - For example, pressure her to become pregnant when she does not want to be or make it difficult for her to get or use FP.
   - Opposition to women's FP use has negative health consequences such as unintended pregnancy and unsafe abortion.
   - Clients experiencing RC can be supported with strategies to use the FP method of their choice that is most convenient or works best for them.

2. Ask the following question to screen for RC:
   - Are you currently experiencing any challenges to your use of family planning from your partner or other family members?

NB: A client who responds YES to this question is POSITIVE for RC.

3. Provide a supportive and validating response to the client, whether they screen RC positive or negative. Do not pressure clients to disclose RC. Affirm client's experiences and inform them that the facility is a safe space for seeking help.

4. Manage clients who screen positive or negative for RC as follows:

RC Positive	RC Negative
• **Thank them for sharing their experience with you emphasizing that you know these issues are difficult to talk about.**<br>• **If positive, inform clients that these experiences are not their fault.**	• Explain that you are pleased to hear this is not happening in their relationship.<br>• Let the client know they can come to the clinic to talk about this and seek help in the future if their situation changes.
• **Discuss strategies to use the FP method of their choice that is most convenient or works best for them.**<br>• **Provide educational mini-booklet on Family Planning, RC and IPV.**	• Provide educational mini-booklet on Family Planning, RC and IPV.
• **Offer to provide male partner engagement and FP counseling. Never pressure client to bring male partner.**	• Offer to provide male partner engagement and FP counseling. Never pressure client to bring male partner.

5. Return to BCS+ Algorithm.

MINISTRY OF HEALTH

INTIMATE PARTNER VIOLENCE (IPV) SCREENING & SUPPORT

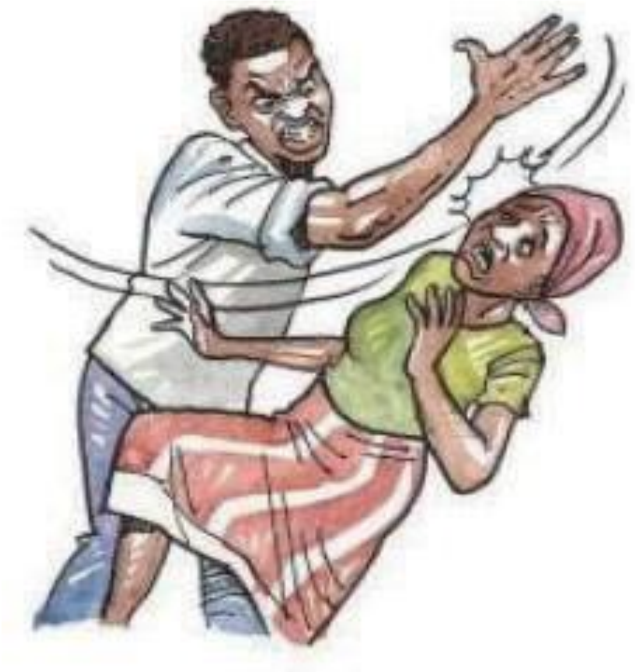

MINISTRY OF HEALTH

INTIMATE PARTNER VIOLENCE (IPV)
SCREENING & SUPPORT

Take the time to assess the client's risk for intimate partner violence and the potential risks that accompany FP use.

1. Begin by sharing some information on intimate partner violence with the client:
    - Many clients are treated badly by their partners, and it is important for healthcare providers to discuss these experiences with all of their clients.
    - There are support services available for those experiencing IPV, and the healthcare provider can link clients to these services if the client so wishes.
    - Some examples of IPV are: being physically hurt by a partner, being screamed at or treated badly by a partner, or being forced to do something sexual that you do not like or do now want to do.

Ask the client:
Are you currently experiencing anything like this from your partner?

NB: A client who responds YES to the question is POSITIVE for IPV.

2. Provide a supportive and validating response to the client, whether they screen positive or negative. Do not pressure clients to disclose IPV. Affirm client's experiences and inform them that the facility is a safe space for seeking help.

3. Manage clients who screen positive or negative for IPV as follows:

- **If POSITIVE for IPV:**
    - o Support the client and validate their experience.
    - o Counsel the client on available support services and offer warm referral to the service(s) of their choice.
- **If NEGATIVE for IPV:**
    - o Explain that you are pleased to hear this is not happening in their relationship.
    - o Assure the client that they can always come back to the clinic to receive support if they experience IPV in the future or refer anyone experiencing IPV.
- **If client does not respond to screening question or is unsure:**
    - o Assure the client that they can always come back to the clinic to receive support if they experience IPV in the future or refer anyone experiencing IPV.
