# Supplementary material for: Strengthening Kenya's public health response to reproductive coercion and intimate partner violence in family planning clinics: applying the FRAME + IS approach
Source: Front Reprod Health. 2026 Jan 5;7:1630877. doi: 10.3389/frph.2025.1630877 (PMC12813199; doi:10.3389/frph.2025.1630877)
Supplement: Supplementary file 6 [file Datasheet5.pdf]

MY BODY, MY RIGHT

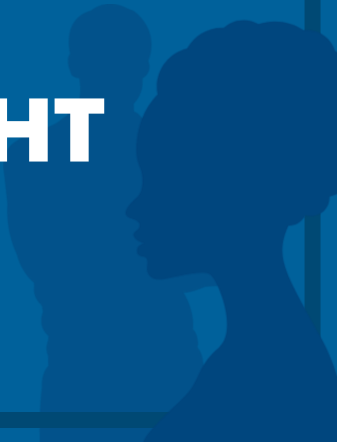

Some women feel pressured to become pregnant or find it difficult to use a contraceptive method in their relationship.

Ask yourself...

► Have you ever felt pressured or forced to become pregnant?

► Are you treated poorly because you do not wish to become pregnant, you wish to use a contraceptive method, or you wish to end a pregnancy?

► Have you ever had your contraceptive method taken away or destroyed?

► Have you ever been forced to use a contraceptive method when you didn't want to?

► Have you ever been prevented from accessing contraception or abortion care?

► Have you ever been forced to end a pregnancy when you wanted to remain pregnant?

If you answered yes, you are not alone.

Help is available:

[Insert health facility name here]

[Insert health facility phone number here]

You have the **RIGHT** to choose your contraceptive method.

There are methods you can use without **ANYONE** knowing!

Your health provider can help you find the method that works best for you.

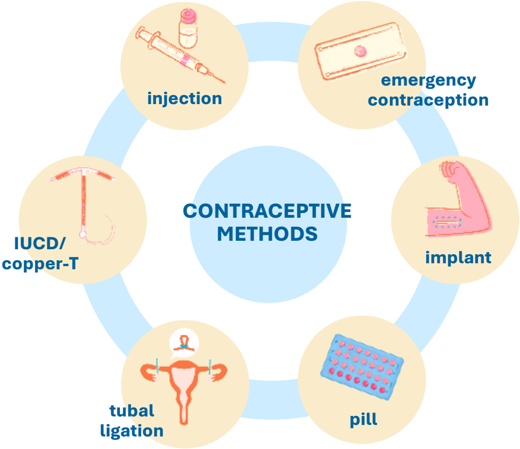

SAFE | EFFECTIVE | PRIVATE

Worldwide, nearly 1 in 3 women have experienced physical or sexual abuse by their intimate partner.

Ask yourself...

► Are you in a relationship where you are threatened, frightened, insulted, or treated badly?

► Are you in a relationship where you are being physically hurt?

► Are you in a relationship where you feel pressured or forced to have sex or do something sexual that makes you uncomfortable?

If you answered yes, you are not alone.

Help is available:

[Insert IPV support service name here]

[Insert IPV support service phone number here]

[Insert IPV support service name here]

[Insert IPV support service phone number here]

ARCHES

EMPOWERING WOMEN TO CONTROL THEIR REPRODUCTIVE HEALTH

Use the QR code to access a digital copy of this booklet.
